# Hydroxyapatite composited PEEK with 3D porous surface enhances osteoblast differentiation through mediating NO by macrophage

**DOI:** 10.1093/rb/rbab076

**Published:** 2021-12-16

**Authors:** Xingdan Liu, Liping Ouyang, Lan Chen, Yuqin Qiao, Xiaohan Ma, Guohua Xu, Xuanyong Liu

**Affiliations:** 1 State Key Laboratory of High Performance Ceramics and Superfine Microstructure, Shanghai Institute of Ceramics, Chinese Academy of Sciences, Dingxi Road 1295, Shanghai 200050, China; 2 Center of Materials Science and Optoelectronics Engineering, University of Chinese Academy of Sciences, Yuquan Road 19, Beijing 100049, China; 3 Tongren Hospital, Shanghai Jiao Tong University School of Medicine, 1111 Xianxia Road, Shanghai 200336, China; 4 School of Materials Science, and Engineering & Henan Key Laboratory of Advanced Magnesium Alloy & Key Laboratory of Materials Processing and Mold Technology (Ministry of Education), Zhengzhou University, Science Avenue 100, Zhengzhou 450001, China; 5 Cixi Center of Biomaterials Surface Engineering, Shanghai Institute of Ceramics, Chinese Academy of Sciences, Wenwei Road 345, Ningbo 315300, China; 6 Department of Orthopedic Surgery, Spine Center, Changzheng Hospital, Naval Medical University, No.415 Fengyang Road, Shanghai 200003, China

**Keywords:** polyetheretherketone, three-dimensional porous, hydroxyapatite, osteoimmunology, nitric oxide

## Abstract

The adverse immune response mediated by macrophages is one of the main factors that are prone to lead poor osseointegration of polyetheretherketone (PEEK) implants in clinic. Hence, endowing PEEK with immunomodulatory ability to avoid the adverse immune response becomes a promising strategy to promote bone repair. In this work, sulfonation and hydrothermal treatment were used to fabricate a 3D porous surface on PEEK and hydroxyapatite (HA) composited PEEK. The HA composited PEEK with 3D porous surface inhibited macrophages polarizing to M1 phenotype and downregulated inducible nitric oxide synthase protein expression, which led to a nitric oxide concentration reduction in culture medium of mouse bone marrow mesenchymal stem cells (mBMSCs) under co-culture condition. The decrease of nitric oxide concentration could help to increase bone formation-related *OSX* and *ALP* genes expressions and decrease bone resorption-related *MMP-9* and *MMP-13* genes expressions via cAMP–PKA–RUNX2 pathway in mBMSCs. In summary, the HA composited PEEK with 3D porous surface has the potential to promote osteogenesis of PEEK through immunomodulation, which provides a promising strategy to improve the bone repair ability of PEEK.

## Introduction

Polyetheretherketone (PEEK) is the clinical implant material approved by Food and Drug Administration [1]. It is widely used as skull repair material, interbody fusion cage, hip joint and spinal implant due to its good biocompatibility, mechanical properties, physicochemical properties, easy processing and so on. However, PEEK is prone to poor osseointegration and hinders bone repair because of its biological inertness. Although many studies focused on modifying PEEK with good osteogenic performance and realized remarkable properties *in vitro*, the undesirable effects *in vivo* often occurred [2–5]. This is mainly because that the biological effects caused by implants are the result of combined effects of multiple cells. It will go through complex processes, such as immune response, activation of complement system and recruitment of bone cells after materials implanted into the body [6–9]. Especially, the time and intensity of implants triggering immune response play an important role in the process of bone repair [7, 10, 11]. Hence, the design of modified surface with the function of regulating immune response to avoid adverse immune response is an effective strategy to improve osteogenic differentiation of PEEK materials.

As one of the important types of immune cells, macrophages play an essential role in regulating the host immune response. Macrophage is a plasticity and pluripotent cell population with two main phenotypes: pro-inflammatory phenotype (M1) and anti-inflammatory phenotype (M2) [12–14]. Osteoimmunology studies show that there is a tight connection between macrophages and bone cells, which share cytokines, signaling molecules and so on [15, 16]. For example, the pro-inflammatory factors secreted by M1 macrophages, such as tumor necrosis factor-α (TNF-α) and interleukin-1β (IL-1β), can enhance osteoclast activity and inhibit osteoblast differentiation. M2 phenotype can secret cytokines, such as transforming growth factor-β (TGF-β) and interleukin-4 (IL-4), to induce osteoblast migration, proliferation and extracellular matrix deposition in the early stage of osteogenic differentiation. They can also produce vascular endothelial growth factor (VEGF) and other factors to promote angiogenesis, which is beneficial for new bone formation and remodeling [17–19]. The excessive inflammatory response would disrupt the balance of bone formation and bone resorption, whereas an appropriate immune microenvironment is beneficial for osteogenic differentiation and blood vessel formation [12, 16, 20]. Therefore, taking macrophages as the target cells and modifying the surface of materials to enable them inhibiting M1 phenotype while promoting the transformation of anti-inflammatory M2 phenotype is an effective way to promote the rapid regression of inflammation, thus enhancing bone repair of implants.

Numerous studies have found that physical and chemical properties of biomaterials surface (e.g. morphology [21], elastic modulus [22], element composition [13] or surface functional group [7]) can regulate immune response and tissue healing through affecting cells adhesion, migration and polarization to regulate immune response and tissue healing. The morphological characteristics of materials, especially the design of surface with micro/nano-morphological features is one of the valuable strategies to improve the biological function of materials [20, 21]. For example,poly (lactic-co-glycolic acid) (PLGA) and bioactive glass-based scaffolds with macro–micro–meso pores could increase M2 polarization, and enhance the angiogenesis and osteogenic performance compared with the scaffolds with macro or macro–meso pores [23]. The 3D porous structure on the sulfonated PEEK could reduce infiltration of inflammatory cells compared with PEEK [24]. In addition, the bioactive elements on the surface also play an important role in the macrophage polarization and bone healing [9, 25, 26]. For instance, tricalcium phosphate with submicron surface topography could promote M2 macrophages polarization [27]. Nano-hydroxyapatite (HA)/PEEK coated implants could reduce inflammation and promote osseointegration in a periodontitis model [28]. Calcium is a key element of regulating the immune response [29, 30]. A lot of reports suggested that extracellular calcium could influence its influx via the calcium channels to modulate macrophage polarization, such as further regulating phosphorylation of protein kinase C β [31]. Therefore, the modified surface with both structure and chemical elements may play synergistic effects to realize superior osteogenesis ability of implants through regulating immune response than that with single modified surface [32]. The internal mechanism among the materials, macrophages and bone cells based on the design needs to be further studied [9].

Consequently, we constructed a surface with both physical and chemical signals on PEEK surface. HA was blended with PEEK powders to obtain HA composited PEEK (PHA) in this work. The sulfonation and hydrothermal treatments were applied to construct a 3D porous structure on PEEK and PHA surfaces. The immune response of macrophages regulated by materials and the immunomodulation effects on the osteogenic performance of mouse bone marrow mesenchymal stem cells (mBMSCs) in the co-culture condition were researched. The interaction mechanism among implants, macrophages and mBMSCs was further studied.

## Materials and methods

### Sample preparation

Medical grade PEEK and PHA (30% wt%) sheets (10 mm × 10 mm × 1 mm) were polished on one side to be the front of the samples. Subsequently, the samples were ultrasonically cleaned in order with acetone, ethanol and ultrapure water, and dried naturally for use. The pretreated samples were named PEEK and PHA, respectively. In order to obtain a uniform 3D porous structure, PEEK and PHA samples were sulfonated by immersing in concentrated sulfuric acid (95–98%), and the liquid was magnetically stirred at room temperature. Next, the samples were soaked in ultrapure water for 10 min to remove residual concentrated sulfuric acid. Then the samples went through hydrothermal treatment with ultrapure water at 120°C for 4 h to remove excess sulfur, washed with ultrapure water and dried at room temperature. The sulfonated PEEK and PHA samples were denoted as SP and SPHA, respectively. The samples were treated with ultraviolet radiation overnight before biological experiments.

### Surface characterization

#### Surface structure and chemical characterization

Field-emission scanning electron microscopy (Magellan 400, FEI, USA) was used to observe the surface structure of samples, and the surface element content and distribution were characterized with the energy dispersive X-ray spectroscopy (EDS; SDD550, IXRF, USA). X-ray diffraction (XRD; D8 Discover, Germany) was used to detect the phase composition of samples.

#### Contact angle analysis

The contact angle test (Automatic Contact Angle Meter Model SL200B, Solon, China) was used to evaluate the surface wettability of samples. At room temperature, 2 μl of ultrapure water was dropped on the surface of samples. Then, the pictures of water droplets were taken by the camera after stabilization, and the contact angle value was read by the device. Three points were tested for each group of samples.

#### Ion release

PHA and SPHA samples were immersed in 10 ml of 0.9% NaCl aqueous solution, and then placed in a 37°C incubator. The soaking solution of 1, 3, 5, 7, 14, 21 and 28 days were collected and the calcium ion concentration was determined by inductively coupled plasma optical emission spectrometer (Vista MPX, NYSE: A, USA).

### Inflammatory responses of macrophages

#### Cell culture

The mouse-derived mononuclear macrophage leukemia cells (RAW264.7; cells were kindly provided by Cell Bank, Chinese Academy of Sciences, Shanghai, China) were seeded in the cell culture flasks, and maintained inDulbecco's modified eagle medium (DMEM) (high glucose; Gibco, USA) medium with 10% fetal bovine serum (Gibco, USA) and 1% penicillin and streptomycin (Antibiotic-Antimycotic; Gibco, USA) in a 5% CO_2_ humidified atmosphere at 37°C. The cells were passaged every 3 days by a ratio of 1:3.

#### Cell viability and morphology

Macrophages were seeded on different samples with a density of 1 × 10^5^ cells per well and cultured in 24-well plates for 4 h, 1 day and 4 days. Cell viability was evaluated by alamarBlue™ (Thermo Fisher Scientific Inc., USA) assay. At every point in time, each well was added 500 μl of fresh full culture medium with 10% alamarBlue™ and incubated for another 2 h in the dark. Then, 100 μl of the solution was taken out and added into a 96-well black plate. The fluorescence intensity was detected at an excitation wavelength of 560 nm and an emission wavelength of 590 nm by a microplate reader (Cytation5, USA) to calculate the cell proliferation. Four samples of each group were tested. At each time point, the samples were taken out to observe cells morphologies. The cells were rinsed twice with phosphate buffered saline (PBS) for 5 min and fixed with 500 μl of 2.5% glutaraldehyde solution in the dark overnight. Then, a series of ethanol solutions (30, 50, 75, 90, 95 and 100% v/v) and the hexamethyldisilazane-ethanol solutions (1:2, 1:1 and 2:1 v/v) were used to dehydrate the cells for 10 min sequentially. After drying, the cells were sprayed with platinum, and the cell morphology was observed by scanning electron microscopy (S-3400N TypeI, Hitachi, Japan).

#### Immunofluorescence staining

The marker protein of different macrophage phenotypes was fluorescently labeled by antigen–antibody binding. The surface fluorescence density was tested to evaluate the macrophage phenotype on various samples. Macrophages were seeded on the surface of the samples with a density of 1 × 10^5^ cells per well at 37°C and cultured for 4 days. After washed by PBS, the cells were fixed with 4% paraformaldehyde at 4°C in the dark. After permeabilized for 2 min with 0.1% (v/v) Triton X-100, 1% wt% BSA (Sigma-Aldrich, USA) was added to block the Fc-receptor. Then, the cells were incubated with CD206 (Abcam, UK, 1:50) and inducible nitric oxide synthase (iNOS) (Novus, USA, 1:50) antibody at 4°C overnight. Afterwards, cells were incubated with donkey anti-mouse secondary antibody IgG H&L Alexa Fluor 594 (Abcam, UK, 1:200) and donkey anti-rabbit IgG H&L Alexa Fluor 488 (Invitrogen, Thermo Fisher Scientific Inc., USA, 1:200) for 2 h in the dark. After washed with PBS,4',6-diamidino-2-phenylindole (DAPI) was used to stain the nuclei in the dark at room temperature for 10 min. Confocal laser scanning microscope (Leica TCS SP8, Germany) was used for observation, and the fluorescence data were quantitatively analyzed using Image J software.

#### Flow cytometry

The proportions of M1/M2 phenotypes of macrophages on the sample surface were tested by flow cytometry. Five samples of each group were placed in each well in a 6-well plate, and macrophages were seeded with a density of 6 × 10^5^ cells per well. They were incubated at 37°C for 4 days. After that, the cells were digested with 0.05% Ethylene diamine tetraacetic acid (EDTA)-trypsin and collected. After washed and resuspended with PBS, the cells were incubated with phycoerythrin-conjugated anti-mouse F4/80 antibody (Thermo Fisher Scientific, USA), Alexa Fluor 488-conjugeted anti-mouse CD206 antibody (Thermo Fisher Scientific, USA) and CCR7 (Bioss, Beijing, China) at 4°C for 30 min. After washed again, the cells were transferred intoFluorescence activated cell sorting (FACS) tubes and a flow cytometry (CytoFLEX, Beckman) was used to detect 10 000 events per tube.

#### Cytokine secretion assays

The concentration of inflammation-related cytokines released by macrophages on samples was tested. Macrophages were seeded with a density of 1 × 10^5^ cells per well and cultured for 1 and 4 days. Subsequently, the culture solution was collected and used to test the concentration of the pro-inflammatory cytokines, interleukin-6 (IL-6), TNF-α and the anti-inflammatory cytokines TGF-β, IL-4, IL-10 according to the protocol by Enzyme-linked immunosorbent assay. The concentration of the released cytokines was calculated from a standard curve. And the iNOS in medium culture with cells for 1 and 4 days was tested by nitric oxide (NO) synthase kit (Nanjing Jiancheng Bioengineering Institute, China).

#### Real-time polymerase chain reaction analysis

The expression of inflammation-related gene in macrophages on the surface of samples was quantitatively analyzed by real-time polymerase chain reaction (RT-PCR). Macrophages were seeded in 24-well plates with a density of 1 × 10^5^ cells per well on samples for 4 days. The total RNA was extracted from cells by TRIzol^TM^ Reagent (Invitrogen, Thermo Fisher Scientific Inc., USA). One microgram of RNA was taken to synthesize the complementary DNA (cDNA) with the Transcriptor First-Strand cDNA Synthesis Kit (Roche, Switzerland) according to the protocol. Glyceraldehyde-3-phosphate dehydrogenase (GAPDH) was used as housekeeping gene, and the relative expression of target genes was tested by using the 2^−ΔΔCt^ analysis method. RT-PCR was carried out by LightCycler^®^ 480 System (Roche, Switzerland). All of primers were purchased from BioTNT and listed in Supplementary Table S1.

### Effects of immunomodulation on mBMSCs

#### Cell culture

The mBMSCs (cells were provided by Shanghai Zhong Qiao Xin Zhou Biotechnology Co., Ltd, Shanghai, China) were used to evaluate the osteogenesis activity. The cells were seeded in the T25 culture flasks, and cultured with mesenchymal stem cell medium (Sciencell Research Laboratories, Inc., USA) in a 5% CO_2_ incubator at 37°C. The cells were passaged at a ratio of 1:3 every 3 days, and the experiments were conducted with mBMSCs at passages 2–5.

#### Co-culture condition of mBMSCs

**Figure 1.**
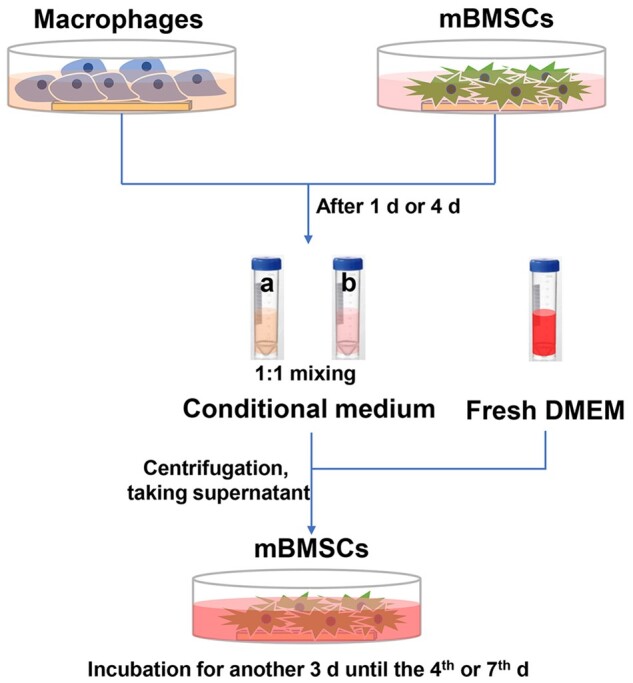
Schematic diagram of co-culture process

Macrophages and mBMSCs were separately seeded on the samples with DMEM full medium mentioned in ‘Cell culture’ section. After 1 or 4 days, the medium cultured with macrophages and mBMSCs was collected and mixed in a ratio of 1:1. Centrifugation at 1400 rpm was used to remove the dead cells from the conditional medium. The conditional medium collected at 1 and 4 days were used to culture mBMSCs for 4 and 7 days. The schematic diagram of mBMSCs cultured with conditional medium was shown in Fig. 1.

#### NO concentration in medium

Macrophages were seeded on samples at a cell density of 1 × 10^5^ cells per well. The mBMSCs were seeded on samples at a cell density of 1 × 10^4^ cells per well for 4 days and a cell density of 5 × 10^3^ cells per well for 7 days. The conditioned medium was added after culturing separately for 1 or 4 days, and then the mBMSCs on samples were cultured for another 3 days according to the methods described in ‘Co-culture condition of mBMSCs’ section. The culture medium was collected after 4 and 7 days. The NO assay kit (Beyotime, China) was used to test NO concentration in the cells culture environment and the operation was according to the protocol.

#### Osteogenesis-related genes expressions under co-culture condition

Macrophages were seeded on the samples at a density of 1 × 10^5^ cells per well and that mBMSCs at a cell density of 2 × 10^4^ cells per well. After 1 or 4 days, the conditioned medium was added to culture the mBMSCs for another 3 days. Then, the cells were lysed with Trizol^TM^ reagent, and the relative expression of genes in the cAMP pathway and osteogenesis was tested by RT-PCR. The β-actin was used as a housekeeping gene, and the test methods was the same as described in ‘Real-time polymerase chain reaction analysis’ section. The primers of genes were listed in Supplementary Table S2.

### Statistical analysis

All data are expressed as mean ± standard deviation and analyzed by GraphPad Prism 5. Statistically significant differences (*P*) are calculated by one-way analysis of variance (ANOVA), two-way ANOVA and Tukey’s multiple comparison tests. A value of *P *<* *0.05 was considered statically significant and was represented by the symbol ‘*’, a value of *P *<* *0.01 was represented by ‘**’, *P *<* *0.001 was ‘***’ and *P *<* *0.0001 was ‘****’.

## Results

### Surface characterization

**Figure 2.**
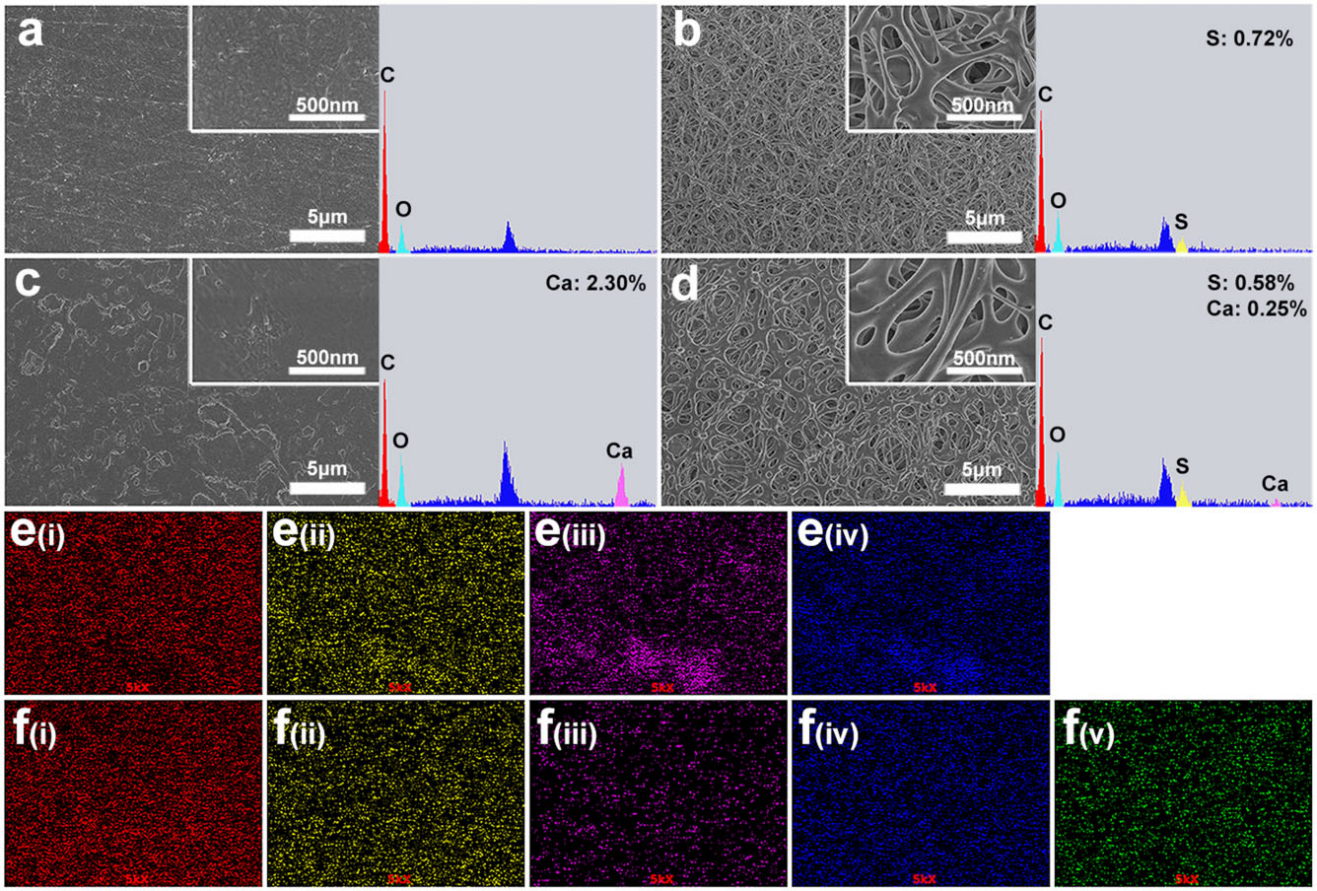
Surface morphologies and elemental characterization of different samples scanning electron microscopy and EDS spectra of PEEK (**a**); SP (**b**); PHA (**c**); and SPHA (**d**). EDS mapping images of PHA (**e**); SPHA (**f**) (i, ii, iii, iv and v represent C, O, Ca, P and S elements, respectively).

Surface structure and element composition of the samples were shown in Fig. 2. PEEK and PHA samples were smooth and flat, while SP and SPHA samples showed uniform and similar 3D porous structures. The EDS spectrum showed that the sulfur content of SP and SPHA samples were 0.72% and 0.58%, respectively (Fig. 2a–d). The calcium content of the PHA sample was significantly higher than that of SPHA, and they were 2.30% and 0.25%, respectively. In addition, Fig. 2e and f shows that the elements on the surface of PHA and SPHA samples were uniformly distributed. It was worth noting that calcium distribution on the surface of SPHA samples was more uniform than that on the surface of PHA samples.

**Figure 3.**
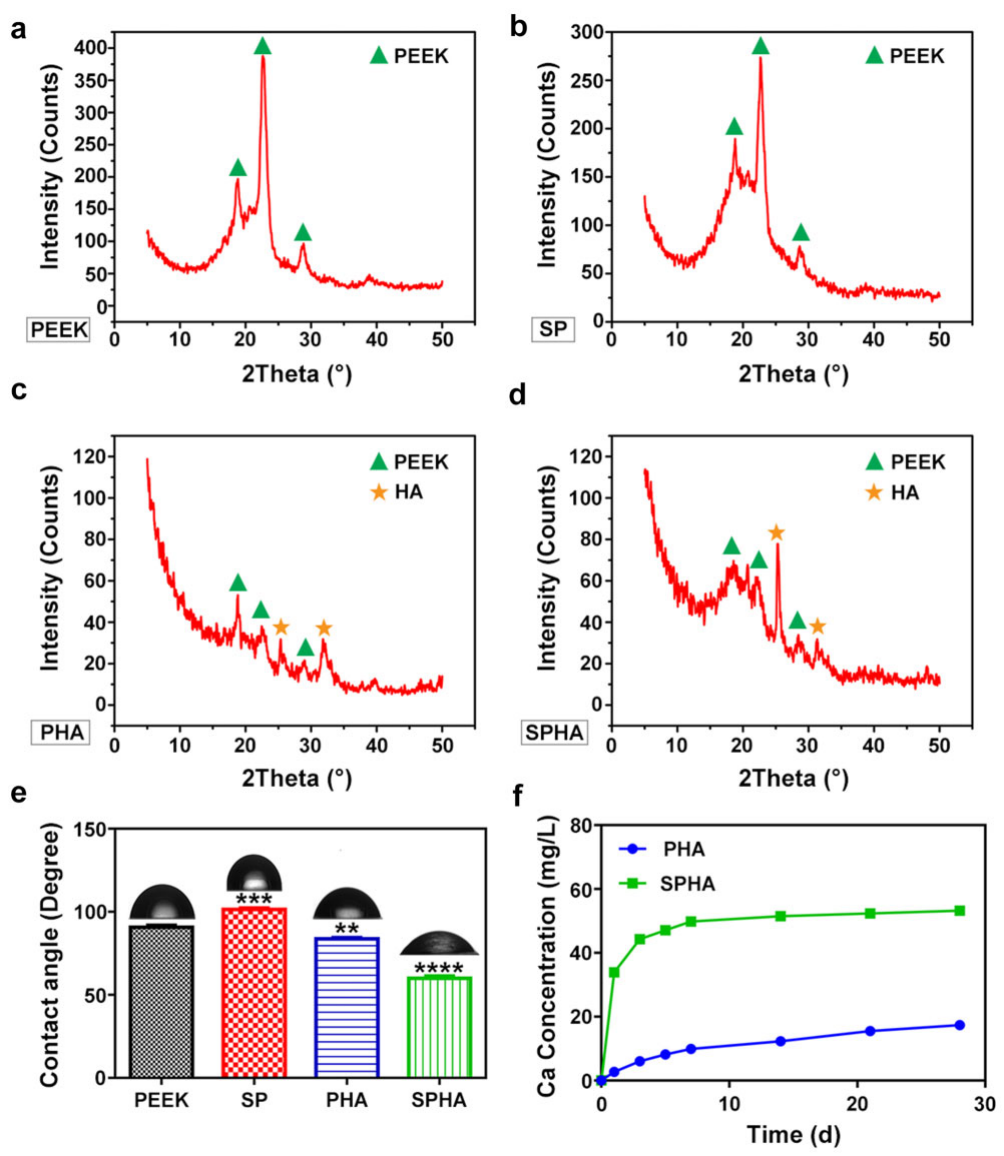
Characterization of the surface properties of samples. XRD spectra of PEEK (**a**); SP (**b**); PHA (**c**); and SPHA (**d**). Surface contact angles of samples (**e**). Ca^2+^ concentrations of PHA and SPHA groups immersed in the normal saline solution for 1, 3, 5, 7, 14, 21 and 28 days (**f**).

XRD test was conducted to determine the phases after chemical treatment, and the spectra were shown in Fig. 3a–d. The results showed that PEEK and SP samples had three peaks at 18.86°, 22.84° and 28.64°, which corresponded to the characteristic peaks of PEEK. PHA and SPHA samples also had peaks at 25.40° and 31.82° except for the peaks of PEEK, which corresponded to the characteristic peaks of HA [33]. The contact angles of sample surfaces were tested and the results were shown in Fig. 3e. The surface contact angles of PEEK and SP samples were 90.72° and 101.38°, respectively, appearing hydrophobic. The contact angles of PHA and SPHA samples with the addition of active substances were 83.73° and 59.98°, respectively, which showed that the surface of SPHA sample was hydrophilic. Ca is an important functional element and involved in many processes *in vivo*. The Ca^2+^ release rules of PHA and SPHA sample surfaces in the normal saline were tested (Fig. 3f). These two groups of samples rapidly released Ca^2+^ within 7 days, and the release rate gradually slowed down after 7 days. The total amount of Ca^2+^ released from the SPHA sample within 28 days was 53.21 mg/l, while that of the PHA sample was 17.37 mg/l. The amount of Ca^2+^ released from the SPHA sample was about three times higher than that of the PHA sample.

### Immune response of macrophages

#### Cell activity and morphology

**Figure 4.**
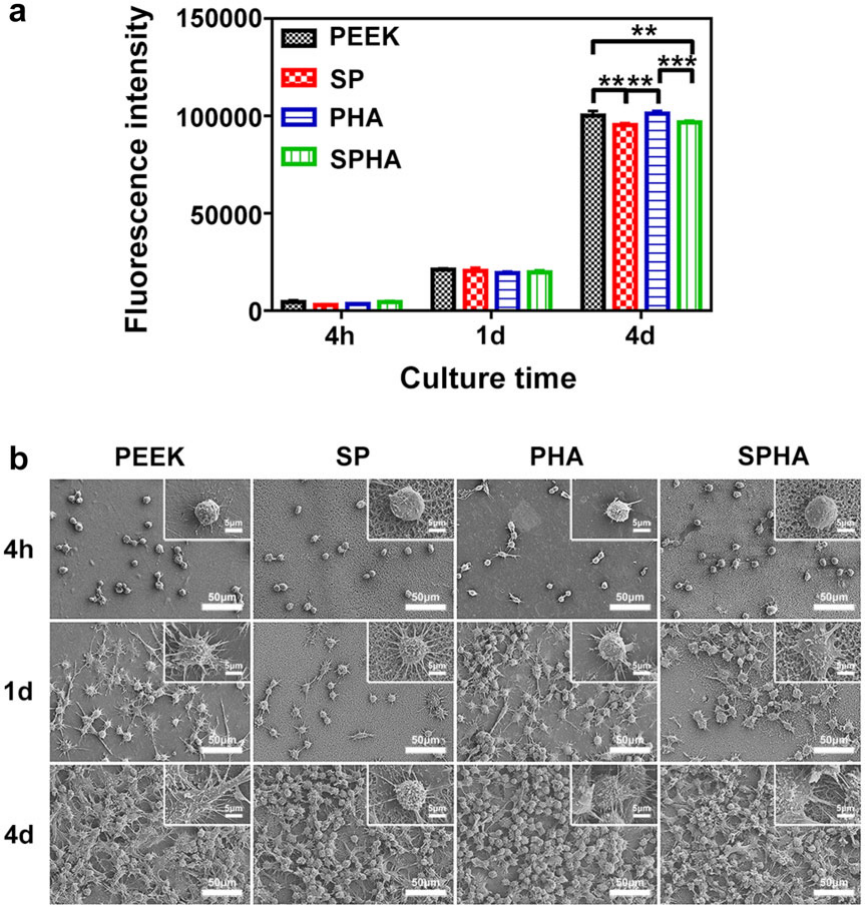
The macrophages activities on various samples. Cell proliferation (**a**); and morphologies of RAW264.7 cultured on sample surfaces at low and high magnifications (**b**).

Macrophages are the main immune cells and mediate the immune response induced by biomaterials. In this work, the RAW264.7 cell line was used to evaluate the inflammatory response on the surface of the materials. In order to study the activity of macrophages, the proliferation and morphologies of cells cultured on the samples were tested, and the results were shown in Fig. 4. After cultured for 4 h and 1 day, there was no significant difference in the proliferation of the cells among the four groups. Cell proliferation of PEEK and PHA were significantly higher than that of SP and SPHA groups after cultured for 4 days (Fig. 4a). Figure 4b showed that the morphologies of cells cultured on the samples. After cultured for 4 h, all of the cells appeared to be a spherical shape. After 1 day, the cells spread out with pseudopodia in different forms, and those on the surface of SPHA sample showed largest spread area. After 4 days, the formed macrophages biofilms can be observed on each group. The proliferation and adhesion of macrophages cultured on the samples surface at the later culture time were enhanced compared with that at the previous culture time, which indicates that all samples show well biocompatibility to macrophages.

#### Polarization of macrophages

**Figure 5.**
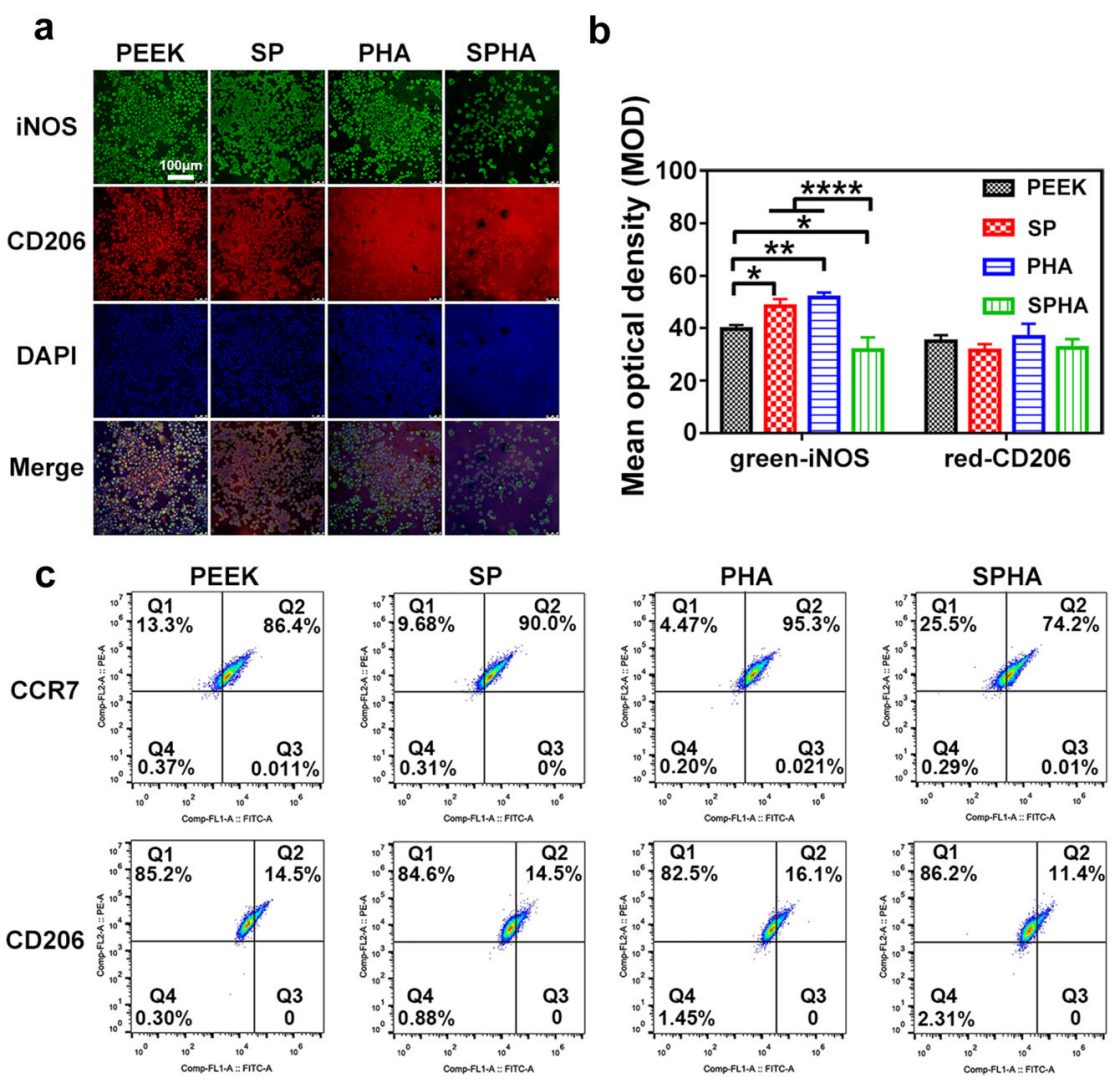
Immunofluorescence staining images of RAW264.7 cultured on samples for 4 days (**a**) and corresponding quantitative analysis (**b**); iNOS (green) was selected as a marker for M1 macrophages, CD206 (red) was selected as a marker for M2 macrophages and the nuclei was stained by DAPI (blue). Flow cytometry analysis of RAW264.7 cultured on samples after 4 days (**c**).

Immunofluorescence staining and flow cytometry were used to analyze macrophages polarization, which closely related to the inflammatory response. iNOS and CD206 were used to label M1 with green and M2 with red, respectively. The polarization of stained macrophages cultured on the sample was observed and the results were shown in Fig. 5a. The trend of green fluorescence intensity of the cells cultured on various samples was as follows: PHA ≈ SP > PEEK > SPHA, and the trend of red fluorescence intensity was as follows: PHA > PEEK ≈ SP > SPHA. The mean optical density results analyzed by Image J software showed that M2 macrophages had similar polarization ratio on each group, but the polarization trend of M1 macrophages on various samples had significant difference: PHA ≈ SP > PEEK > SPHA (Fig. 5b). The green fluorescence intensity of macrophages on the SPHA sample was significantly reduced, while those on PHA and SP samples were enhanced compared with the PEEK sample. Flow cytometry was used to analyze the relative proportion of macrophage phenotypes. F4/80, CCR7 and CD206 were selected as the marker of macrophages, M1 phenotype and M2 phenotype. As shown in Fig. 5c, Q2 area represents M1 macrophage (F4/80^+^/CCR7^+^) or M2 macrophage (F4/80^+^/CD206^+^) in the two sets of graphs. The proportions of M1 macrophages on PEEK, SP, PHA and SPHA were: 86.40, 90.00, 95.30 and 74.20%, respectively; the proportions of M2 macrophages on PEEK, SP, PHA and SPHA were: 14.50, 14.50, 16.10 and 11.40%, respectively. The overall trend of macrophage polarization was similar to that of immunofluorescence staining. These results indicated that SPHA could significantly reduce M1 macrophages ratio, which was facilitate for tissue repair.

#### Cytokines and genes expressions

**Figure 6.**
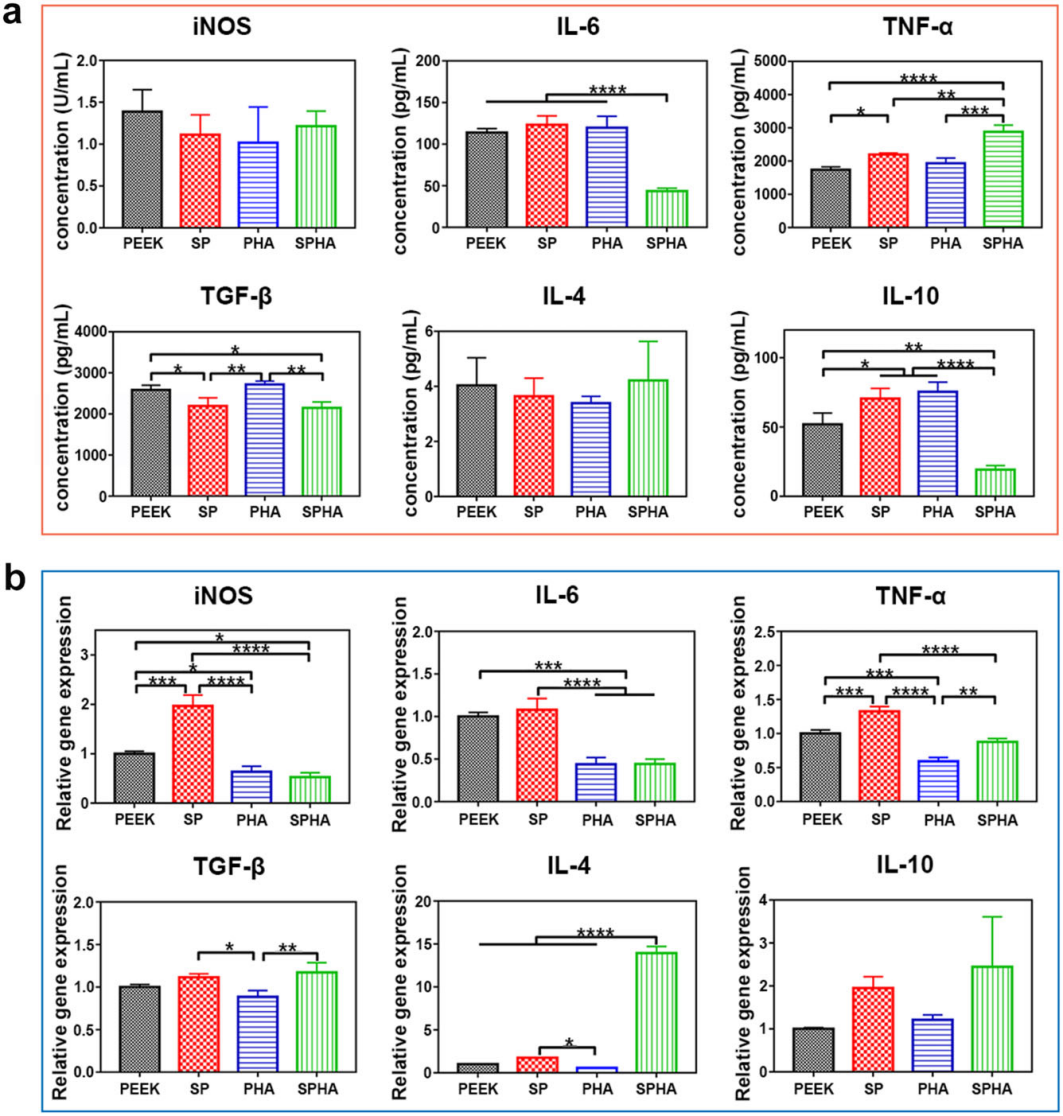
Immune response of macrophages on different samples. Inflammatory cytokines concentration detected by enzyme-linked immunosorbent assay after cultured for 4 days (**a**); inflammation-related genes expressions in macrophages cultured on each group of samples for 4 days (**b**).

The cytokines’ concentration in the medium of macrophages cultured on samples for 4 days was tested to study the inflammatory response of macrophages, and the results were shown in Fig. 6a. The concentration of iNOS in the medium of macrophages on SP, PHA and SPHA samples was less than that of PEEK. The SPHA sample significantly reduced the IL-6 concentration secreted by macrophages, while there was no significant difference among the other three groups. The TNF-α concentration in medium of macrophages could be significantly increased in SP and SPHA groups compared with that of cells in PEEK and PHA groups. As for the anti-inflammatory cytokines, SP and SPHA samples reduced TGF-β concentration secreted by macrophages. There were no significant differences in the regulating IL-4 secretion among the groups, and the SPHA sample could slightly increase its concentration in medium of macrophages. Besides, the SPHA sample significantly inhibited the secretion of IL-10 among the four groups.

In order to evaluate the inflammatory response induced by samples, the expressions of inflammatory-related genes in macrophages were further tested by RT-PCR (Fig. 6b). The results showed that PHA and SPHA samples containing bioactive component significantly reduced the expressions of *iNOS*, *IL-6* and *TNF-α* genes in macrophages, while the SP sample with the 3D porous structure upregulated the expression of iNOS and TNF-α compared with PEEK sample. There were different effects between SP and SPHA samples on regulating *iNOS* and *TNF-α* genes expressions in macrophages, which indicated the bioactive component on the SPHA sample surface could effectively downregulate their expression. In terms of the anti-inflammatory gene expression, SP and SPHA samples could promote the expressions of *TGF-β, IL-4* and *IL-10* genes; the PHA sample could promote the expression of *IL-10* gene in macrophages, and the effects of the PHA sample on regulating *TGF-β* and *IL-4* genes in macrophages were similar to PEEK sample. Among them, the SPHA sample has the most significant effect on promoting of anti-inflammatory genes expressions.

### Osteogenic activity of mBMSCs mediated by NO

#### NO concentration in medium

**Figure 7.**
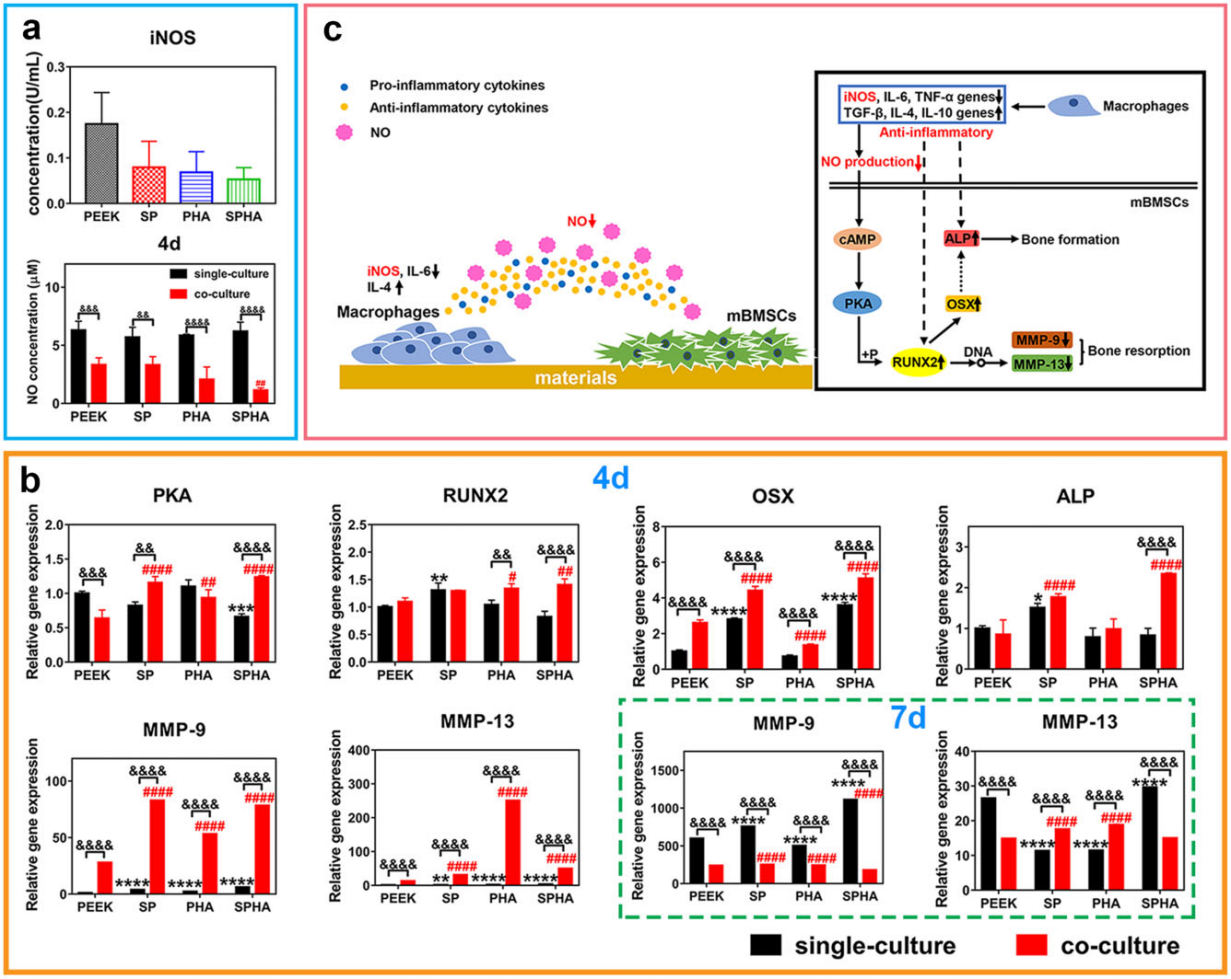
Influence of immune regulation stimulated by materials on the composition in culture environment and osteogenic differentiation of mBMSCs. iNOS concentration in the medium of macrophages cultured on materials for 1 day and NO concentration in the culture environment of mBMSCs under single culture and co-culture at 4 days (**a**); osteogenesis-related genes expressions in mBMSCs (**b**). ‘green dotted box’ shows the genes expression at 7 days and the others are at 4 days. ‘black *’ and ‘red #’ were vs PEEK in single culture group and co-culture group, respectively; ‘&’ was vs the single culture group at the same sample group. Schematic diagram of the interaction among materials, macrophages and mBMSCs (**c**).

NO is a gaseous reactive nitrogen species, which plays an essential role in regulating cell functions and transmitting important messages *in vivo* [34]. The NO concentration in the medium of mBMSCs (denoted by single culture group) as well as mBMSCs co-cultured with macrophages (denoted by co-culture group) on the samples were tested to study the influence of materials on the mBMSCs behaviors by adjusting intermediate media in medium after stimulating macrophages. As shown in Fig. 7a, NO concentration in the medium of mBMSCs with co-culture was reduced compared with that in single culture group at 4 days due to the iNOS concentration variation in the conditional medium collected from macrophages at 1 day. And NO concentration in SPHA group was reduced compared with the other three groups in co-culture group at 4 days. Their NO concentration was lower than 5 μM, and SPHA group had the lowest NO concentration, which was 1.13 μM. There was no significant difference in the NO concentration among four groups in the single culture group at 4 days, and their concentration were about 5–10 μM.

#### Osteogenesis-related genes expressions

Studies have shown that NO plays an important role in regulating the proliferation, matrix secretion and apoptosis of osteoblasts, the formation and maturation of osteoclasts as well as maintaining the balance between bone formation and bone resorption [34, 35]. The expressions of osteogenesis-related genes were carried out by RT-PCR, and the results were shown in Fig. 7b. In the single culture group, SPHA and SP with 3D porous structure significantly downregulated PKA and upregulated osterix (*OSX*) gene expression, while SP also upregulated *RUNX2* and *ALP* genes expression compared with PEEK at 4 days. After culturing mBMSCs with the 1-day conditional medium for 4 days, SPHA upregulated *RUNX2* gene expression, while both SPHA and SP upregulated *PKA*, *OSX*, *ALP* genes expression compared with PEEK. The PHA group could upregulate *PKA*, *RUNX2*, genes, but downregulate *OSX* gene expression compared with PEEK. On the other hand, the other three groups upregulated matrix metalloproteinase *(MMP)-9* and *MMP-13* genes expression compared with PEEK group at 4 days under both single and co-culture conditions. When the mBMSCs were cultured on samples for 7 days, SPHA and SP upregulated *MMP-9* gene expression, while PHA downregulated its expression compared with PEEK in the single culture group. And SPHA upregulated *MMP-13* gene expression, while SP and PHA downregulated its expression compared with PEEK under single culture condition. In the co-culture group for 7 days, SPHA downregulated *MMP-9* and *MMP-13* genes expression compared with SP and PHA. Compared with the other groups, the mBMSCs in SPHA group has better osteogenesis performance under co-culture. Figure 7c showed the scheme of the SPHA sample promoting bone healing through stimulating macrophages. In detail, the SPHA sample could reduce NO concentration in medium of mBMSCs through decreasing iNOS protein expression from macrophages to upregulate RUNX2 expression, which upregulated *OSX* and *ALP* genes to promote bone formation and downregulated *MMP-9* and *MMP-13* genes expression in mBMSCs to inhibit bone resorption.

## Discussion

Biomaterials would quickly trigger the host immune response when implants enter in the body. Excessive inflammatory responses would impede osseointegration by forming a thick fibrous encapsulation around implants [4, 6]. Recently, it has become a future trend to endow biomaterials with immunomodulatory properties through surface modification to promote bone repair [36, 37]. Sulfonation is an effective means to activate PEEK surface [38, 39]. In this work, PEEK and PHA are sulfonated and hydrothermally treated to improve their biological properties. SPHA has both superior surface properties and bioactive element for cells response. Even though the structure of SPHA is similar to SP, the water contact angle of SPHA was significantly decreased due to the HA exposure. The total amount of Ca^2+^ released from the SPHA sample is significantly higher than that from the PHA sample due to the exposed HA wrapped by PEEK after sulfonation, which is facilitated for improving Ca^2+^ release of SPHA sample surface (Fig. 3). Therefore, Ca^2+^ concentration of PHA was higher than PEEK but lower than SPHA.

Macrophages are the important immune cells, and their polarization state is closely related to affect wound healing. More and more evidences indicate that the polarization state of macrophages is highly sensitive to the physical and chemical properties of biomaterials [21, 40]. In this work, all samples show noncytotoxic to the macrophages. Cells cultured on the SPHA sample have the largest spread area due to the exposed HA after sulfonation (Fig. 4). Different polarization phenotypes of macrophages determine their function in the process of bone repair [13]. Excessive activation of M1 macrophages would result in a prolonged pro-inflammatory stage, which would lead to tissue damage and chronic inflammatory states [25, 41]. In detail, M1 macrophages excessively secrete a variety of pro-inflammatory cytokines (such as TNF-α, IL-1β, etc.) and produce a large amount of reactive oxygen species, which can mediate MMP secretion and osteoclast differentiation, thereby impairing tissue regeneration and wound healing [17, 42]. SP and PHA group increased the M1 macrophages ratio, while the SPHA group reduced its ratio compared with the PEEK sample (Fig. 5). The concentration of Ca^2+^ and surface properties are both attributed to M1 macrophages ratio. On the one hand, the inflammation response to Ca^2+^ depending on the concentration. Ca^2+^ can enhance inflammation through noncanonical Wnt5A/Ca^2+^, while higher extracellular Ca^2+^ can reduce inflammation through activating the calcium-sensing receptor signal cascade to inhibit NF-κB, TNF-α expression as well as the Wnt5a/Ror2 signaling pathway [25]. Therefore, PHA shows an inferior M1 inhibition than PEEK and SPHA. On the other hand, surface properties also contribute to the immune regulation. Macrophages tend to concentrate on the surface with small pores due to their inability to penetrate, which leads an increased M1 ratio [43]. Therefore, 3D porous structure on the SP sample surface enhanced the M1 macrophages ratio compared with the PEEK group. SPHA is more hydrophilic than SP, which leads to a suppressed M1 proportion [7]. Combined with high Ca^2+^ concentration and surface properties endow SPHA a superior M1 suppression than PEEK, SP and PHA groups. M2 macrophages can increase the IL-10, TGF-β, insulin-like growth factor-1 and *VEGF* genes expressions, and inhibit the pro-inflammatory genes expressions to alleviate or eliminate inflammation, thereby promoting bone formation [44, 45]. There are four M2 macrophage subtypes: M2a, M2b, M2c and M2d. They are induced by different factors and perform different functions. M2a can produce IL-4 and downregulate the secretion of pro-inflammatory factors, such as IL-6. Especially, it does not express iNOS; M2b macrophages can secrete high levels of IL-10 and TNF-α at the same time; M2c macrophages can be induced by IL-10, TGF-β, etc.; and M2d can secret IL-10 and VEGF to promote angiogenesis [19]. Compared to the other three groups, the SPHA sample upregulates anti-inflammatory genes expressions and promotes IL-4 secretion in macrophages, indicating that the SPHA sample may mainly induce the M2a subtype of macrophages, which is closely related to tissue healing (Fig. 6).

Many studies have reported that the immune system interacts strongly with the skeletal system through the paracrine [15, 16].iNOS not only induce inflammation, but also regulate the production of NO by acting on the substrate L-arginine [46]. NO, as a second messenger and neurotransmitter, is a highly dispersive free radical that plays an important regulator role in cell-to-cell information transmission. It is also an important signaling molecule in the process of bone remodeling, acting on osteoclasts and osteoblasts to maintain the balance of bone metabolism [34]. SPHA can reduce NO production of macrophages by inhibiting iNOS protein expression. Macrophages on the SPHA sample may tend to be activated to M2a, which could significantly downregulate iNOS expression. And furthermore, the NO concentration was reduced in the mBMSCs culture environment under co-culture condition. Studies have shown that the concentration of NO is closely related to the activity of osteoclasts and plays a dual role in regulating their activity [47]. In our work, the lower concentration of NO (<5 μM) in medium could improve the osteogenesis and bone resorption while the medium concentration (10–20 μM) could inhibit bone resorption for the SPHA group in the co-culture condition (Fig. 7 and Supplementary Figs S2 and S3).

NO could affect bone formation and bone resorption via activating cAMP/PKA, cGMP/PKG and MAPK pathways [48, 49]. In our study, the SPHA sample could promote adhesion, proliferation, spreading and osteogenic differentiation performance of mBMSCs (Supplementary Fig. S1). SPHA could upregulate *PKA*, *RUNX2*, *OSX* and *ALP* genes expressions in mBMSCs at 4 days, and downregulate *MMP-9* and *MMP-13* genes expression at 7 days compared with SP and PHA sample under the co-culture condition (Fig. 7 and Supplementary Fig. S3). And the relative ALP activity could also be improved for the SPHA group through co-culture at 7 days (Supplementary Fig. S4). Many reports have suggested that RUNX2 is an important cytokine for early osteogenic differentiation [50, 51]. On the one hand, RUNX2 can promote bone formation by influencing osteogenic differentiation indicators, such as ALP through its downstream gene OSX [52, 53]. On the other hand, MMPs play a fundamental role in the process of mineralized matrix degradation that could lead to bone resorption, and the mechanism of their expression largely depends on the activation of osteogenic differentiation-specific transcription factors, such as RUNX2 [34]. The SPHA sample could promote osteogenic differentiation in early stage and inhibit bone resorption in later stage through regulating RUNX2 expression in mBMSCs via cAMP–PKA signaling pathway. In addition, the SPHA sample shows anti-inflammatory effect in macrophages, which is conducive to promote bone formation and tissue repair [54–56]. In summary, the SPHA sample mainly could regulate the RUNX2 expression *via* cAMP–PKA pathway in mBMSCs to promote bone formation in early stage and inhibit bone resorption in later stage through downregulating the iNOS protein expression from macrophages to reduce the NO concentration in medium.

## Conclusion

In this work, a surface with 3D porous structure and bioactive element (Ca) was constructed by sulfonating and hydrothermally treating the PHA. Its effects on inflammation, osteogenesis and the molecular mechanism of osteoimmunology were studied *in vitro*. The results of materials-mediated immune response showed that PHA with 3D porous surface could significantly reduce the proportion of M1 macrophages, inhibit expressions of pro-inflammatory genes and promote those of anti-inflammatory genes. The PHA with 3D porous surface could reduce NO production by significantly downregulating the iNOS protein expression in macrophages. The complex surface could further increase RUNX2 gene expression *via* cAMP–PKA pathway, thereby upregulating osteogenesis-related genes, OSX and ALP expression in early stage and downregulating MMP-9 and MMP-13 genes expression in later stage in mBMSCs cultured in conditional medium collected from macrophages. This work provides a modification method to construct a surface on PEEK to regulate the inflammatory response thereby promoting the bone repair, which is of great significance for promoting the clinical application of PEEK materials.

## Supplementary data

Supplementary data are available at *REGBIO* online.

## Funding

Financial support from the National Natural Science Foundation of China (U21A20100, 81772363, 32000938) , Science and Technology Commission of Shanghai Municipality, China (20ZR1465000), High-end Entrepreneurial and Innovative Teams of Ningbo High-level Talents Project (2018A-09-C), Shenzhen Science and Technology Funding(JCYJ20210324120009026), and S&T Innovation 2025 Major Special Program of Ningbo (2018B10040) are acknowledged.

*Conflict of interest statement*. All authors declare that there are no conflicts of interest.

## Supplementary Material

rbab076_Supplementary_Data
