# Supplementary material for: Hydroxyapatite composited PEEK with 3D porous surface enhances osteoblast differentiation through mediating NO by macrophage
Source: Regen Biomater. 2021 Dec 16;9:rbab076. doi: 10.1093/rb/rbab076 (PMC9039504; doi:10.1093/rb/rbab076)
Supplement: rbab076_Supplementary_Data [file rbab076_supplementary_data.docx]

**Hydroxyapatite composited PEEK with 3D porous surface enhances osteoblast differentiation through mediating NO by macrophage**

Xingdan Liu ^1, 2^, Liping Ouyang ^3, *^, Lan Chen ^4^, Yuqin Qiao ^1, 2^, Xiaohan Ma ^1, 5^, Guohua Xu ^6, *^, Xuanyong Liu ^1, 2, 5 *^

^1^ State Key Laboratory of High Performance Ceramics and Superfine Microstructure, Shanghai Institute of Ceramics, Chinese Academy of Sciences, Shanghai 200050, China

^2^ Center of Materials Science and Optoelectronics Engineering, University of Chinese Academy of Sciences, Beijing 100049, China

^3^ Hongqiao International Institute of Medicine, Shanghai Jiao Tong University School of Medicine, Shanghai 200336, China

^4^ School of Materials Science and Engineering & Henan Key Laboratory of Advanced Magnesium Alloy & Key Laboratory of Materials Processing and Mold Technology (Ministry of Education), Zhengzhou University, Zhengzhou 450001, China

^5^ Cixi Center of Biomaterials Surface Engineering, Shanghai Institute of Ceramics, Chinese Academy of Sciences, Ningbo 315300, China

^6^ Department of Orthopedic Surgery, Spine Center, Changzheng Hospital, Naval Medical University, No.415 Fengyang Road, Shanghai 200003, People’s Republic of China

* Corresponding Author:

Prof. Xuanyong Liu, Dr. Liping Ouyang, and Guohua Xu

Tel.: +86 21 5241 2409. Fax: +86 21 5241 2409.

E-mail: [xyliu@mail.sic.ac.cn](mailto:xyliu@mail.sic.ac.cn),

[lpouyang91@163.com](mailto:lpouyang91@163.com)

xuguohuamail@smmu.edu.cn

**1. Materials and methods**

1.1. Cell adhesion assay

The mBMSCs were seed at a density of 5×10^4^ cells per well and cultured for 1, 4 and 24 h. At each incubation time, the cells were fixed with 4% paraformaldehyde solution. Then they were permeated with Triton X-100 reagents for 2 min and blocked with BSA afterwards. The cytoskeletons were stained with fluorescein isothiocyanate (FITC)-phalloidin (Sigma, USA) for 1 h at room temperature. DAPI was used to stain the cell nucleus for 5 min. The state of cells adhesion was observed under the confocal laser scanning microscope (Leica TCS SP8, Germany).

1.2. Cell viability and morphology

The mBMSCs with a density of 5×10^4^ cells per well were seed on different samples (three replicates) in 24-well plates for 1, 4 and 7 d, and their proliferation was evaluated by the alamarBlue™ assay as described in section 2.3.2. Uniformly, the cells were fixed with the 2.5% glutaraldehyde solution and dehydrated by a series of ethanol solutions and HMDS ethanol solutions consecutively for morphology observation by SEM.

1.3. ALP activity assay

To test the ALP activity, the mBMSCs were cultured at a cell density of 5×10^3^ cells per wells density on samples, while macrophages were seeded on the samples at a density of 1 × 10^5^ cells per well. The methods of co-culture were described in section 2.4.2. Then, the cells on each group of samples (four replicates) were lysed by Lysis buffer mixed solution for 1 h and then incubated with p-nitrophenyl phosphate for 30 min at 37°C. After incubation, the reaction was stopped by adding 1 M NaOH and the absorbance at 405 nm was measured using the microplate reader. The ALP concentration of each group was calculated according to the standard curve. Subsequently, the total protein concentration of each group was obtained by bicinchoninic acid kit (Thermo Fisher Scientific Inc., USA). Finally, the relative ALP activity was analyzed by the ratio of ALP to total protein concentration.

**2. Results**

2.1. The adhesion and viability of mBMSCs with single culture

In the early stage of the interaction between the materials and the cells, the cells would gradually recruit to the surface of the materials, and the materials may affect the cell spread state in the process ^1^. Figure S1a shown the initial adhesion topography of mBMSCs in the single culture condition. After cultured for 1 h, the mBMSCs adsorbed on the surfaces of various samples, showing spherical. After cultured for 4 h, the cells on the surfaces with 3D porous structure tended to spread along the structure skeleton. After 24 h, the cells formed biofilms and extended along the pore skeleton on the surface of SP and SPHA samples. The porous structure could provide a 3D attachment point for mBMSCs and cause their morphologies to be gradually stretched.

The proliferation and morphologies of mBMSCs cultured on the different samples were observed to study their biocompatibility. The proliferation of mBMSCs was shown in Figure S1b. The mBMSCs cultured on each group of samples had good cells activity, and the cells cultured on SP and SPHA samples were better than those on PHA and PEEK samples. Figure S1c showed the cells morphologies cultured on samples. After cultured for 1 d, the mBMSCs cultured on the SPHA sample had the largest spread area. After cultured for 4 d, the cells cultured on each group have formed biofilms. After 7 d, the biofilms became denser. The properties, 3D porous structure and bioactive element (Ca), have shown synergistic effects on promoting cells proliferation and adhesion.


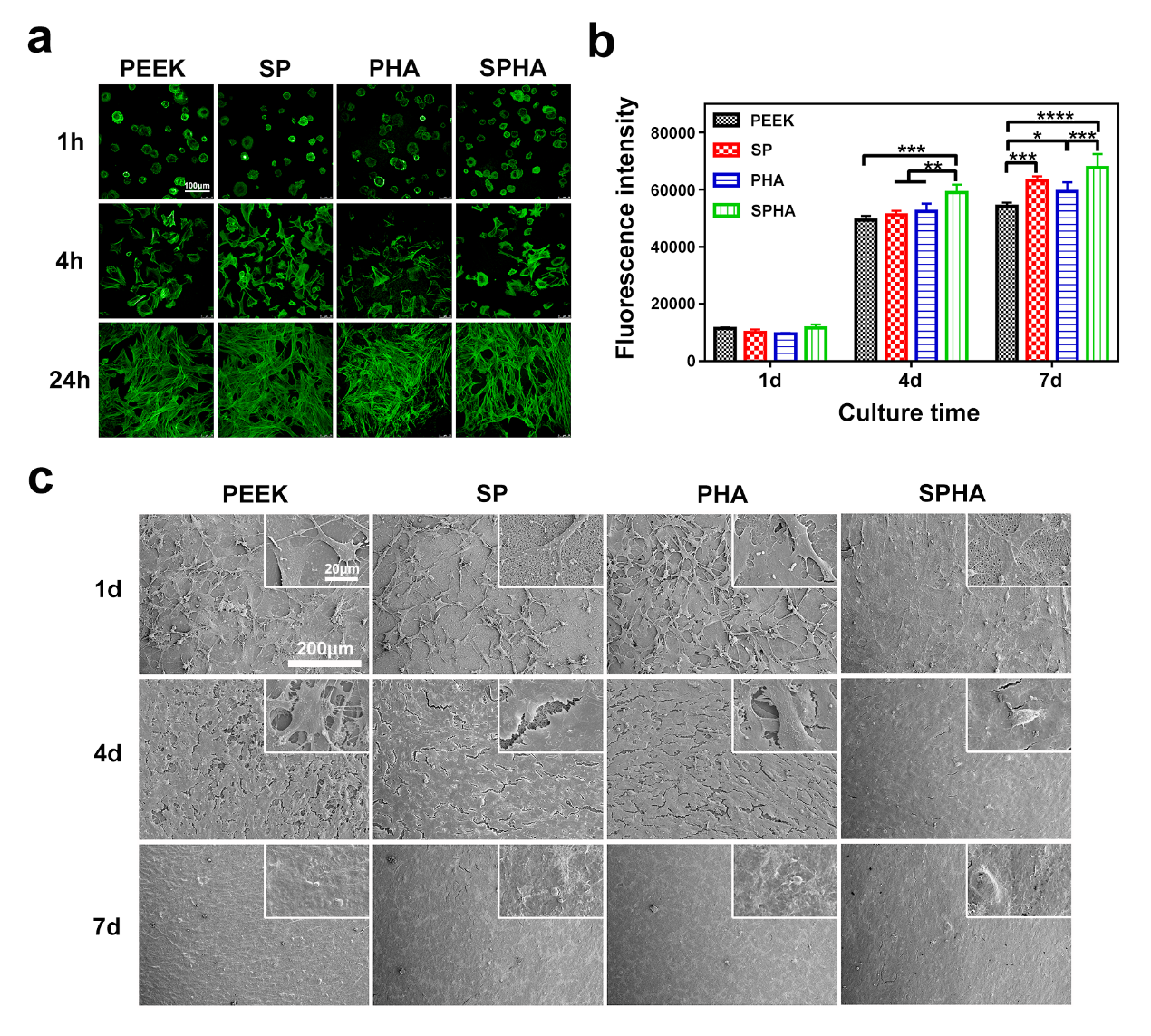


Figure S1. Cytoskeleton staining images of mBMSCs cultured on various samples for 1 h, 4 h, and 24 h (a); Cell proliferation (b) and SEM images (c) of mBMSCs cultured on different samples for 1, 4, and 7 d in the single culture condition.

2.2. NO concentration in medium

NO concentration in the medium of mBMSCs with co-culture and single culture at 7 d was shown in Figure S2. There was no significant difference in the NO concentration of medium between the single culture group and the co-culture group, except for the SP group. NO concentration in other three group were lower than that in the PEEK group. The NO concentration in the medium of PEEK group was about 40μM, while that in the medium of the other three groups were about 10-20μM whether single culture or co-culture conditions.


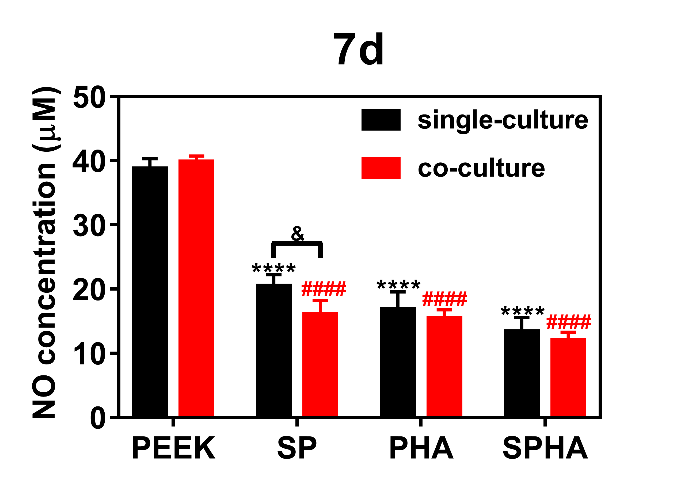


Figure S2. NO concentration in the culture environment of mBMSCs under single culture and co-culture at 7 d.

2.3. Osteogenesis-related genes expressions in the co-culture condition

Figure S3 showed osteogenesis-related genes expressions with co-culture at 7 d. In the single culture group, SP and SPHA group with 3D porous structure down-regulated PKA and RUNX-2 genes expression while the regulation of these genes was reversed in PHA group compared with PEEK group. Compared with PEEK group, the other three groups down-regulated OSX and up-regulated ALP gene expression. After mBMSCs co-cultured for 7 d, the effects among each group on PKA and ALP genes expression were contrary to the results in single culture group, while the influences on RUNX2 and OSX genes expression were similar with the results in single culture group.


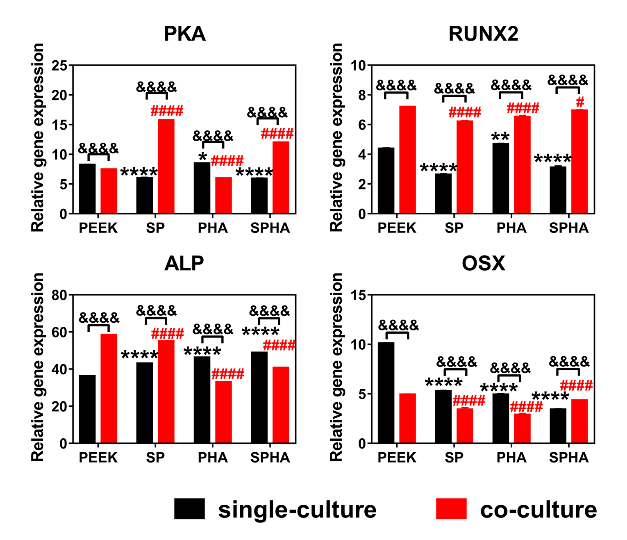


Figure S3. Osteogenesis-related genes expressions in mBMSCs at 7 d. “Black *” and “red *” were versus PEEK in single culture group and co-culture group, respectively; “&” was versus the single culture group at the same sample group.

2.4. Relative ALP activity with co-culture

Alkaline phosphatase (ALP) is a type of marker proteins for osteogenic differentiation. Figure S4 showed the relative ALP activity of mBMSCs with single culture and co-culture. SP and SPHA with 3D porous structure significantly reduced ALP secretion, and the PHA sample also slightly inhibited its secretion compared with PEEK samples under single culture condition. However, there was no significant difference among various samples after co-culture. It indicated that could mitigate the inhibitory effect in single culture by co-culture.


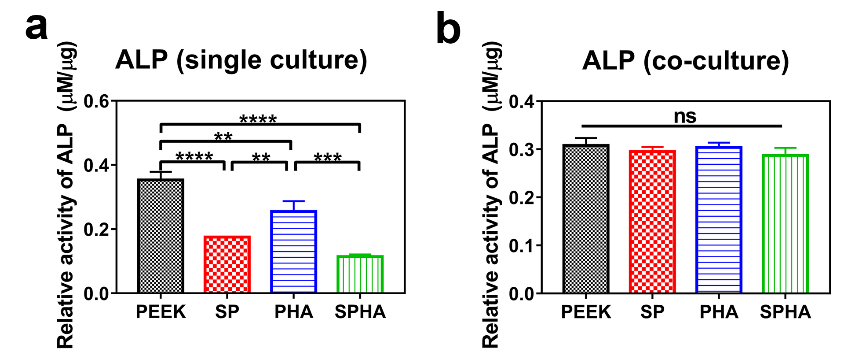


Figure S4. Relative ALP activity in mBMSCs at 7 d under single culture and co-culture.

**Table S1**. Primers used for real time-PCR in the immunological evaluation

| Gene (mouse) | Primer sequences  (F, forward; R, reverse; 5’-3’) | Product size (bp) |
| --- | --- | --- |
| GAPDH | F: GCT CAG GCC TCT GCG CCC T  R: CCT ACT CTC TTG AAT ACC | 115 |
| iNOS | F: TTG ACG CTC GGA ACT GTA  R: GTT GGT GGC ATA AAG TAT GTG | 74 |
| IL-6 | F: CCA AGA GGT AAA AGA TTT AC  R: ATT GAT AAT TTA AAT AAG TA | 161 |
| TNF-α | F: TTA GAG CGG GAT AGT AAC G  R: CAA AAT ACA CAA CAG TGT C | 111 |
| TGF-β | F: AAG GAC CTG GGT TGG AAG T  R: GGT CCT TGC CCT CTA CAA C | 135 |
| IL-4 | F: CCA TGA ATG AGT CCA AGT CC  R: TAA CTT ATG AAT TTT TAA T | 60 |
| IL-10 | F: CCC TTT GCT ATG GTG TCC T  R: GTG GCC AGT TTG TTA TTT AT | 106 |

**Table S2**. Primers used for real time-PCR in the osteogenesis evaluation of mBMSCs

| Gene (mouse) | Primer sequences  (F, forward; R, reverse; 5’-3’) | Product size (bp) |
| --- | --- | --- |
| β-actin | F: CCT CTA TGC CAA CAC AGT  R: AGC CAC CAA TCC ACA CAG | 155 |
| PKA | F: GAT GGA ATG TCT TGT CAG CAT  R: AGT CCT CAC TGC TCT CTA GGC | 103 |
| RUNX2 | F: GCA GCA CGC TAT TAA ATC CAA  R: GCC AAA CAG ACT CAT CCA TTC | 120 |
| OSX | F: CTA GTT CCT ATG CTC CGA CCT  R: GAT GGC AAC GAG TTA ATG AGA | 102 |
| ALP | F: GCA GGC AAG ACA CAG ACT  R: TGG AGG AGA GAA GGT CAG AT | 114 |
| MMP-9 | F: CTG GAA CTC ACA CGA CAT CTT  R: CAC CTT GTT CAC CTC ATT TTG | 100 |
| MMP-13 | F: CCA GAA TAA AGA CTG TGC CAG  R: CCA AGT GTT ACT CGC TAA GGA | 108 |

**References**

1. Shi, Y.; Liu, K.; Zhang, Z.; Tao, X.; Chen, H. Y.; Kingshott, P.; Wang, P. Y., Decoration of Material Surfaces with Complex Physicochemical Signals for Biointerface Applications. *ACS Biomaterials Science & Engineering* **2020,** *6* (4), 1836-1851.
